# Prevalence and Risk Factors of Acute Lower Gastrointestinal Bleeding in Crohn Disease

**DOI:** 10.1097/MD.0000000000000804

**Published:** 2015-05-21

**Authors:** Guanwei Li, Jianan Ren, Gefei Wang, Qin Wu, Guosheng Gu, Huajian Ren, Song Liu, Zhiwu Hong, Ranran Li, Yuan Li, Kun Guo, Xiuwen Wu, Jieshou Li

**Affiliations:** From the Department of Surgery, Jinling Hospital, Medical School of Nanjing University, Nanjing, P.R. China.

## Abstract

Acute lower gastrointestinal bleeding (ALGIB) is a rare but potentially life-threatening complication of Crohn disease (CD). Thus far, few studies of ALGIB in the context of CD have been published, most of which were case reports with limited value. We aimed to explore the prevalence of ALGIB in CD patients, evaluate risk factors for hemorrhagic CD and its recurrence, and analyze clinical data of the death cases.

A total of 1374 CD patients registered from January 2007 to June 2013 were examined. Medical records of 73 patients with ALGIB and 146 matched as controls were reviewed and analyzed retrospectively. Logistic regression and Cox proportional hazards analyses were performed to identify risk factors for ALGIB and the cumulative probability of rebleeding. Kaplan–Meier curves with log-rank tests were used to demonstrate the cumulative survival rates of rebleeding.

The prevalence of ALGIB was 5.31% (73/1374) in this study. In the univariate analysis, possible risk factors for ALGIB were duration of CD (odds ratio [OR] 0.60, 95% confidence interval [CI] 0.33–1.09, *P* = 0.095), perianal disease (OR 1.96, 95% CI 0.92–4.20, *P* = 0.082), left colon involvement (OR 2.16, 95% CI 1.10–4.24, *P* = 0.025), azathioprine use ≥1 year (OR 0.46, 95% CI 0.23–0.90, *P* = 0.023), and previous hemorrhage history (OR 11.86, 95% CI 5.38–26.12, *P* < 0.0001). In the multivariate analysis, left colon involvement (OR 2.26, 95% CI 1.04–4.91, *P* = 0.039), azathioprine use ≥1 year (OR 0.44, 95% CI 0.20–0.99, *P* = 0.044), and previous hemorrhage history (OR 13.04, 95% CI 5.66–30.04, *P* < 0.0001) remained independent influencing factors. Older age (HR 0.23, 95% CI 0.07–0.77, *P* = 0.018), surgical treatment (HR 0.17, 95% CI 0.06–0.50, *P* < 0.001), and having bleeding episodes >3 months ago (HR 0.24, 95% CI 0.07–0.82, *P* = 0.022) resulted to be predictors associated with rebleeding after discharge. Patients who died often suffered severe concomitant diseases, and the overall mortality rate was 8.22% (6/73).

We speculated that a special hemorrhagic phenotype of CD that was predisposed to rebleeding may exist. Further studies are warranted to investigate the pathogenesis and discover the optimum treatments of choice.

## INTRODUCTION

Crohn disease (CD), a subtype of inflammatory bowel disease, is characterized by transmural inflammation involving the full thickness of the bowel disease, which can lead to serious complications including intestinal obstruction, intraabdominal abscesses, and intestinal fistulas. Acute lower gastrointestinal bleeding (ALGIB) is a rare but potentially life-threatening complication of CD, with frequency ranging in various studies from 0% to 6%.^1–8^ Hemorrhagic CD is thus not well known and represents a diagnostic and therapeutic challenge.

Previous studies focusing on ALGIB in the context of CD were scarce, most of which were case reports with a small number of patients. The so far largest study by Kim et al^9^ demonstrated the clinical characteristics and risk factors for ALGIB in Korean patients with CD. However, this study simply investigated the predisposing factors of the hemorrhagic phenotype. In light of the propensity for rebleeding,^9,10^ identifying those at higher risk of future recurrence is essential because intensive therapy and close observation can be given to such patients.

In the present study, we aimed to report the prevalence of ALGIB in Chinese patients with CD and evaluate the risk factors for ALGIB and its recurrence after bleeding stopped. We also indicated the overall mortality rate and analyzed the detailed clinical data of the death cases.

## METHODS

### Patients Inclusion

Medical records of 1374 CD patients, registered in the Department of Surgery of a large tertiary teaching hospital (Jinling Hospital) between January 2007 and June 2013, were retrieved using keywords “gastrointestinal bleeding” or “gastrointestinal hemorrhage.” A total of 73 patients admitted primarily for ALGIB were selected, and their medical history especially previous bleeding episodes were scrutinized. The diagnosis of CD was confirmed based on the clinical, endoscopic, histological, and radiological criteria.^11^ ALGIB was defined as profuse rectal bleeding that required blood transfusions to maintain normal vital signs.^6^ Patients were excluded if the origin of hemorrhage was upper gastrointestinal tract proximal to the ligament of Treitz or anus. Those who suffered gastrointestinal bleeding due to other diseases such as colonic diverticula were also excluded; 146 patients who were admitted for recurrent abdominal pain or diarrhea were matched as controls with the ratio of 2:1.

### Data Management

Medical records were collected and scrutinized retrospectively. Data included sex, date of birth, date of onset of symptoms, disease location, and behavior according to the Montreal classification,^12^ therapeutic modalities, date of final follow-up, and final outcome. Therapeutic modalities were classified as surgical treatment and conservative therapies. Bleeding stop was defined as no further evidence of gastrointestinal bleeding when discharged. This study was approved by the Ethics Committee of Jinling Hospital.

### Statistical Analysis

The statistical analysis was performed using IBM SPSS for Windows version 19.0. Logistic regression was performed to identify risk factors for ALGIB in contrast with the nonbleeding group. Cox proportional hazards regression analysis was utilized in order to examine the independent variables on the cumulative probability of the rebleeding after discharge. Variables in univariate analysis were entered into multivariate analysis if *P* < 0.1, and *P* < 0.05 was considered statistically significant. The method of enter was used in the multivariate analysis. The cumulative rate of rebleeding was manifested by the Kaplan–Meier method and compared by log-rank test. Probability values and confidence intervals were calculated at the 95% level.

## RESULTS

### Prevalence of ALGIB

**FIGURE 1:**
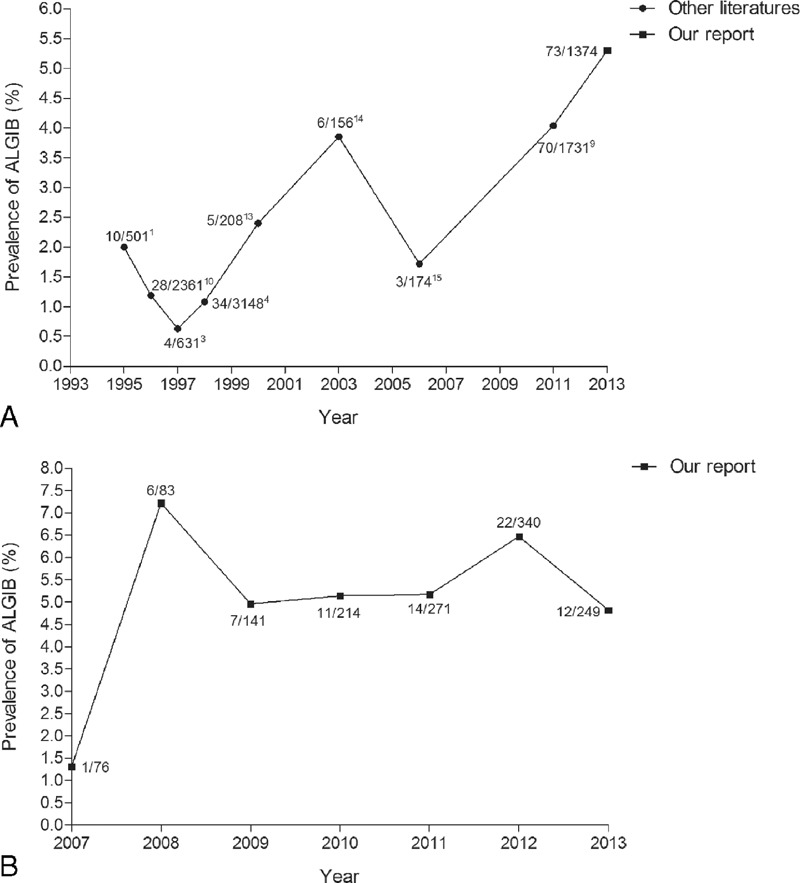
(A) Prevalence of acute lower gastrointestinal bleeding in our report in contrast with other literatures based on the ending year of the study period. Superscript denotes the serial number of literatures. (B) Annual prevalence of acute lower gastrointestinal bleeding in our center from January 2007 to June 2013. Numerator represents the number of cases. Denominator represents the total number of patients. ALGIB = acute lower gastrointestinal bleeding.

The prevalence of ALGIB in our report and other relevant literatures^1,3,4,9,10,13–15^ are shown based on the ending year of the respective study period (Figure 1A). Massive hemorrhage occurred in 0.63% to 5.31% of CD patients, and was most commonly seen in our cohort (73/1374, 5.31%). To note, the annual rate of ALGIB did not show a trend of increase in our center from January 2007 to June 2013 (Figure 1B).

### Risk Factors for ALGIB

Potential risk factors that may have influenced the hemorrhagic form of CD were evaluated by univariate and multivariate logistic regression analysis. In the univariate analysis (Table 1), possible risk factors for ALGIB were duration of CD (OR 0.60, 95% CI 0.33–1.09, *P* = 0.095), perianal disease (OR 1.96, 95% CI 0.92–4.20, *P* = 0.082), left colon involvement (OR 2.16, 95% CI 1.10–4.24, *P* = 0.025), azathioprine use ≥1 year (OR 0.46, 95% CI 0.23–0.90, *P* = 0.023), and previous hemorrhage history (OR 11.86, 95% CI 5.38–26.12, *P* < 0.0001). In the multivariate logistic regression analysis (Table 2), left colon involvement (OR 2.26, 95% CI 1.04–4.91, *P* = 0.039), azathioprine use ≥1 year (OR 0.44, 95% CI 0.20–0.99, *P* = 0.044), and previous hemorrhage history (OR 13.04, 95% CI 5.66–30.04, *P* < 0.0001) remained significant factors that independently affected the risk of ALGIB in CD patients.

**TABLE 1:**
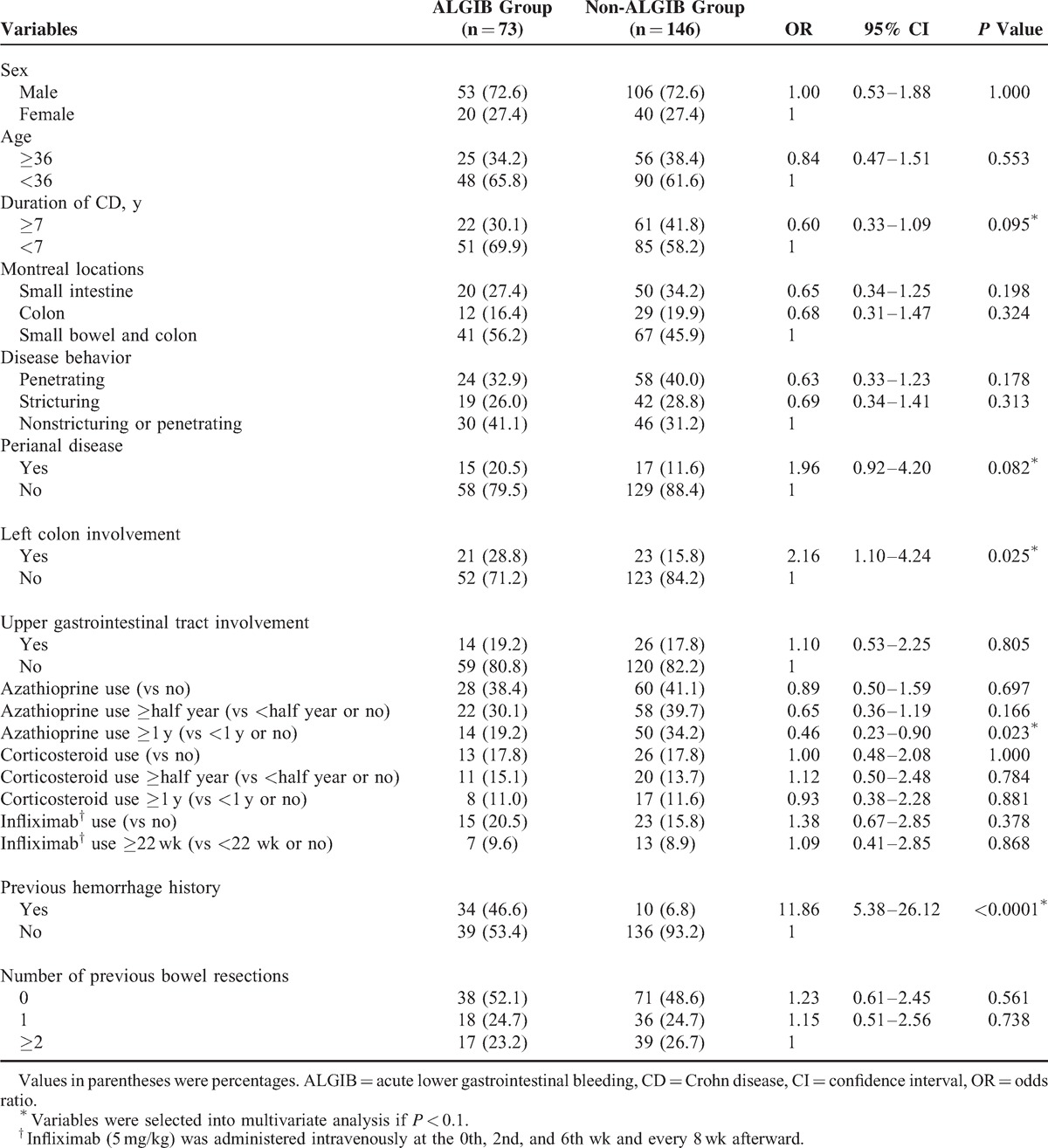
Univariate Analysis of Risk Factors for ALGIB in Patients With Crohn Disease

**TABLE 2:**
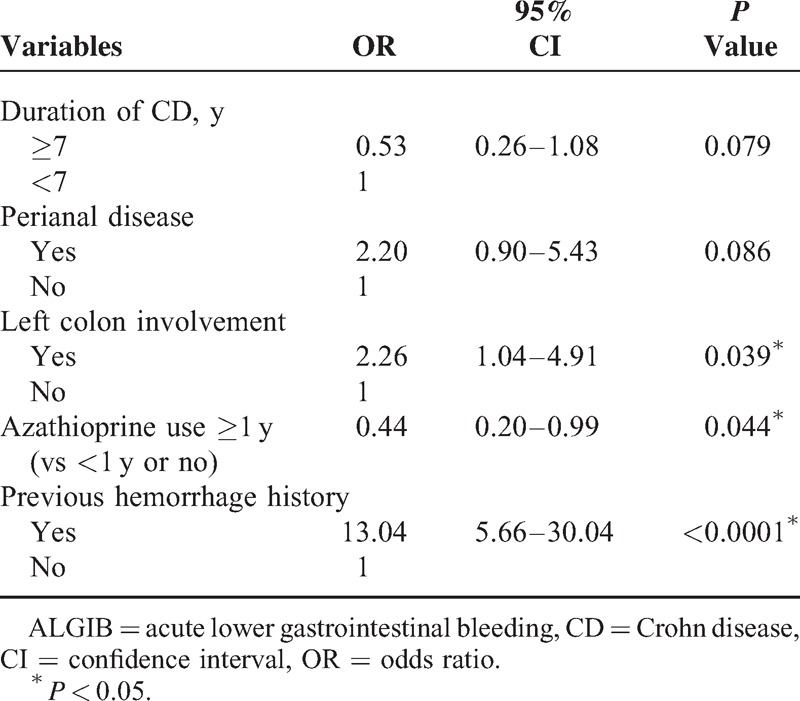
Multivariate Analysis of Risk Factors for ALGIB in Patients With Crohn Disease

### Risk Factors for Rebleeding

Among 73 patients with ALGIB, 36 patients successfully stopped bleeding by medical treatment whereas 29 patients required surgery. Six cases died and the remaining 2 patients were discharged by themselves for economic reasons and lost to follow-up. During the follow-up (10.8 ± 9.5 months) after discharge, lower gastrointestinal hemorrhage recurred in 23 patients (35.4%). Cox proportional hazards regression was then performed to determine the risk factors influencing the cumulative rate of rebleeding among 65 patients (Table 3). Patients aged >36 had a low risk of rebleeding (HR 0.23, 95% CI 0.07–0.77, *P* = 0.018). Surgical treatment significantly reduced the risk of rebleeding (HR 0.17, 95% CI 0.06–0.50, *P* < 0.001). Surgical procedures involved included 12 segmental small intestine resections (41.4%), 7 subtotal or total colectomies (24.2%), 3 segmental colectomies (10.3%), 3 segmental small intestine and colon resections (10.3%), and 4 ileocecectomies (13.8%). Rebleeding rate was significantly reduced in those having bleeding episodes >3 months ago (HR 0.24, 95% CI 0.07–0.82, *P* = 0.022) when stratifying subjects according to bleeding history before admission.

**TABLE 3:**
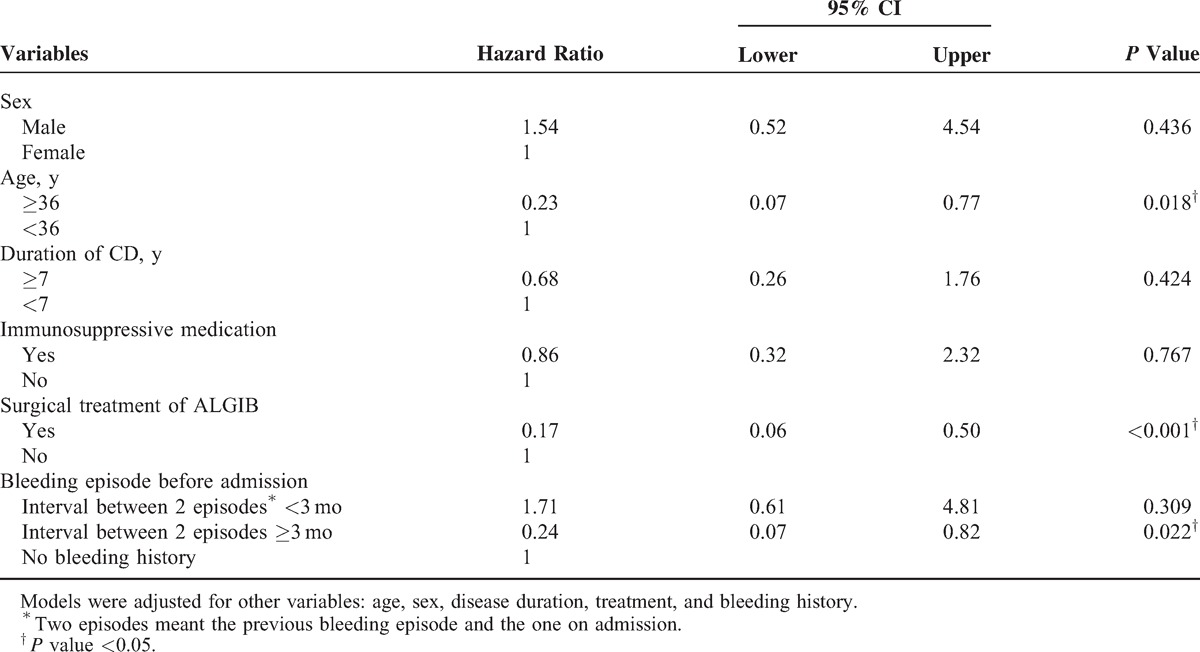
HR of Rebleeding in Patients With Crohn Disease by Multivariate Cox Analysis

### Cumulative Rate of Rebleeding

**FIGURE 2:**
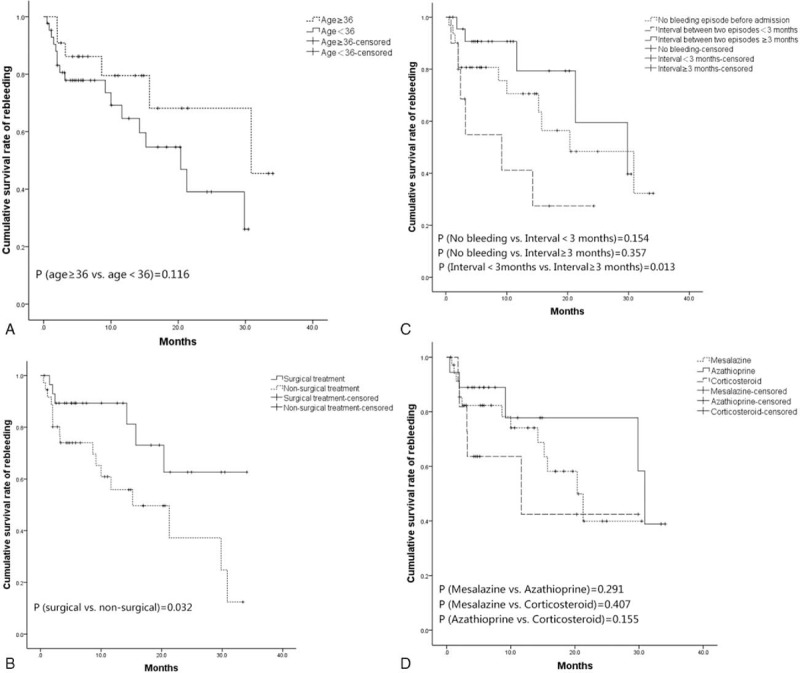
(A) Kaplan–Meier plots illustrating the survival rates of rebleeding in patients aged >36 and <36. The cumulative risk of rebleeding in 2 groups was comparable (*P* = 0.116). (B) Kaplan–Meier plots illustrating the survival rates of rebleeding in patients receiving surgical and nonsurgical treatments. The cumulative risk of rebleeding was significantly lower in the operated patients (*P* = 0.032). (C) Kaplan–Meier plots illustrating the survival rates of rebleeding among patients grouped by the bleeding history before admission. The cumulative risk of rebleeding was significantly higher in patients who had bleeding episodes within the past 3 mo on admission than those with bleeding interval >3 mo (*P* = 0.013). (D) Kaplan–Meier plots illustrating the survival rates of rebleeding among patients receiving mesalazine, azathioprine, and corticosteroid. No specific drug demonstrated a better effect for preventing rebleeding.

The cumulative survival rate of rebleeding among the patients aged >36 was 79.5% after 1 year and 68.1% after 2 years, whereas that of those aged <36 was 64.6% after 1 year and 39% after 2 years (Figure 2A). The cumulative probabilities of recurrence were lower in patients receiving surgeries (*P* = 0.032) (Figure 2B). Cumulative 1 and 2-year survival rates of rebleeding were 89.3% and 62.6%, respectively, in the operated patients whereas 55.8% and 37.2%, respectively, in those receiving conservative therapy. Patients who had bleeding episodes within the past 3 months on admission showed a higher rebleeding rate after bleeding stopped than those with interval >3 months (*P* = 0.013) (Figure 2C). Medication against CD was started after the patients were cured of gastrointestinal bleeding and discharged from hospital. The Kaplan–Meier curve for the rate of rebleeding among the patients receiving mesalazine, azathioprine, and corticosteroid was also shown (Figure 2D). No specific drug demonstrated a better effect for preventing rebleeding.

### Analysis of the Death Cases

Six death cases were observed in the present study, making the mortality 8.22%. Detailed clinical data were retrieved and showed in Table 4. There were 4 males and 2 females, with age ranging from 27 to 47 years; 2 of 6 patients had hemorrhage history on admission, and 5 had previously undergone surgeries. Three patients suffered at least severe leukopenia. Platelet counts were severely low in 4 cases and 3 presented coagulation disorder. Mechanical ventilation was used in 3 of 6 cases. In the respect of comorbidity, the most common concomitant diseases were pneumonia and hydrothorax, especially among the ventilated patients. Notably, 2 patients developed hematological diseases including myelodysplastic syndrome and acute promyelocytic leukemia simultaneously.

**TABLE 4:**
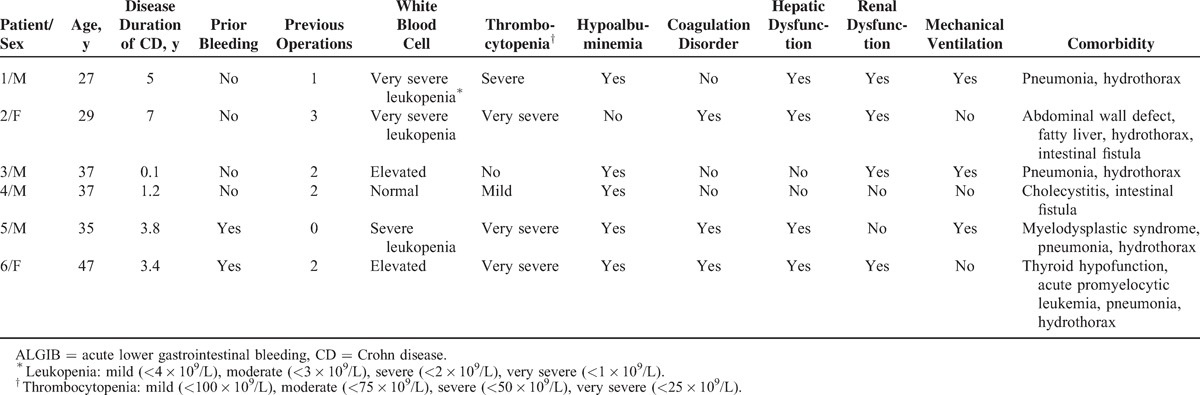
Clinical Data of 6 Death Cases of ALGIB in Patients With Crohn Disease

## DISCUSSION

Massive gastrointestinal hemorrhage is a rare complication of CD. Over a recent 6-year period in our center, ALGIB had been present in only 5.31% of CD patients. The prevalence of ALGIB in the present study was a bit higher than that of other reports.^1,3,4,9,10,13–15^ Varying definitions of severe gastrointestinal bleeding employed by different studies may explain this variation. Therefore, to determine a clear definition of ALGIB will be of significance for future studies, and epidemiological studies are required to explore the actual burden of ALGIB.

There was only 1 study by Kim et al^9^ evaluating risk factors for ALGIB in the context of CD. They manifested that azathioprine/6-mercaptopurine was negatively associated with the risk of bleeding and that may due to the induction of mucosal healing. In our study, azathioprine turned out not to be an independent influencing factor of ALGIB whereas use of azathioprine for >1 year appeared to yield the protective effect. Chatu et al^16^ also reported that CD patients had a significant reduction in the risk of first intestinal surgery when receiving >12 months of thiopurines therapy. Left colon involvement of disease was found to increase the risk of ALGIB. Mechanical wear effect of formed stools in the left colon on colonic lesions may be the explanation. Robert et al^8^ reported a higher frequency of bleeding among the patients with colonic involvement, and Belaiche et al^4^ identified the bleeding site in a colonic ulcer or an ulcerated area in most situations. Another risk factor in our study was previous hemorrhage history, which meant that patients having a prior bleeding episode were prone to suffer another. In fact, hemorrhage did recur in 23 cases (35.4%) during follow-up. Similar high recurrence rates were also reported by many others studies.^4,9,17^ It was therefore surmised that hemorrhagic CD may present a special phenotype of the disease.^14^

To our knowledge, this was so far the first study that evaluated risk factors for bleeding recurrence in the setting of CD. Multivariate analysis showed that older patients (≥36 years) seemed to have a low risk of rebleeding after discharge. The reasons remained unclear. Scarpa et al^18^ demonstrated younger age to be a risk factor for recurrence of stenosis in CD. We also found that the operated patients were at a lower risk of rebleeding than those receiving conservative treatment. Papi et al^17^ reviewed the data of 101 patients from 5 series and drew the same conclusion. In his pooled analyses, recurrence rate of bleeding was significantly higher in conservatively treated patients compared with the operated subjects (38.5% vs 5.7%). That was plausible because the deep mucosal ulcerations, which were supposed to be the origin of ALGIB,^19^ were directly resected in surgical intervention. However, surgeries may result in major complications, such as postoperative bowel obstruction, anastomotic leakages, and fistulas.^20^ Hence, nonsurgical methods are preferred if conditions permit. For example, antitumor necrosis factor-α antibody, which induces rapid mucosal healing,^21,22^ may be an alternative therapy for gastrointestinal bleeding indicated by several case reports.^17,19,23–26^ We failed to find a better preventive effect on rebleeding by the survival analysis among the treatments of mesalazine, azathioprine, and corticosteroid. Infliximab was uncommonly administered to our discharged patients therefore not investigated in the present study due to the health insurance reimbursement policy. The present study also found that patients, who had bleeding episodes within the past 3 months on admission, represented a higher rebleeding rate after bleeding stopped. This observation further supported the hypothesis that there may be a hemorrhagic phenotype of CD, a special cohort predisposed to rebleeding, while overall bleeding rate in CD patients was very low.^14^

Six of 73 (8.22%) patients in this series died. However, overall mortality rates greatly fluctuated because of the rareness of this complication, varying from 0% to 20% among different reports.^1,4,8,9,13–15,27^ In our study, almost all dead patients were complicated by organ dysfunction or comorbidities. Coagulation disorder and the concomitant hematological diseases made it more difficult to palliate the bleeding and leukopenia increased the risk of systemic infection. Therefore, maintaining the organs function and correcting the underlying disorders in time is of even more significance, whereas hemostatic therapy and supportive measures are necessary in the initial management.

In conclusion, we found that patients with prior bleeding history, left colon involvement of disease, and use of azathioprine <1 year were at a higher risk of experiencing ALGIB. This may be useful to help screen the subjects at risk for follow-up care. Older patients seemed to have a low risk of rebleeding after discharge. Surgeries reduced the risk of recurrence but may result in other complications. Therefore, seeking for other avenues of medical treatment to prevent recurrence may be the most important point. Patients with interval <3 months between the last 2 bleeding episodes were subject to a third strike of bleeding. This indicated the presence of a cohort of patients with hemorrhagic phenotype that intensive treatment and close observation should be given to. Severe concomitant diseases were involved in the exacerbation but the overall mortality was relatively low. In a word, further studies are required to investigate the pathogenesis of ALGIB in CD and discover the optimum treatments of choice.
